# Studying alcohol use disorder using *Drosophila melanogaster* in the era of ‘Big Data’

**DOI:** 10.1186/s12993-019-0159-x

**Published:** 2019-04-16

**Authors:** Gregory L. Engel, Kreager Taber, Elizabeth Vinton, Amanda J. Crocker

**Affiliations:** 1 Department of Psychological Sciences, Castleton University, Castleton, VT 05735 USA; 2 Program in Neuroscience, Middlebury College, Middlebury, VT 05753 USA

**Keywords:** Alcohol behavior, Drosophila, Fruit fly, Next-generation sequencing, Animal model

## Abstract

Our understanding of the networks of genes and protein functions involved in Alcohol Use Disorder (AUD) remains incomplete, as do the mechanisms by which these networks lead to AUD phenotypes. The fruit fly (*Drosophila melanogaster*) is an efficient model for functional and mechanistic characterization of the genes involved in alcohol behavior. The fly offers many advantages as a model organism for investigating the molecular and cellular mechanisms of alcohol-related behaviors, and for understanding the underlying neural circuitry driving behaviors, such as locomotor stimulation, sedation, tolerance, and appetitive (reward) learning and memory. Fly researchers are able to use an extensive variety of tools for functional characterization of gene products. To understand how the fly can guide our understanding of AUD in the era of Big Data we will explore these tools, and review some of the gene networks identified in the fly through their use, including chromatin-remodeling, glial, cellular stress, and innate immunity genes. These networks hold great potential as translational drug targets, making it prudent to conduct further research into how these gene mechanisms are involved in alcohol behavior.

## Background

As of 2015, Alcohol Use Disorder (AUD) directly affected over 15 million people in the United States. Its social, financial, and medical repercussions affect countless others [1]. The Center for Disease Control estimates that alcohol use is a contributing factor in over 80,000 deaths annually [2]. The National Epidemiologic Survey on Alcohol and Related Conditions (NESARC) recently found that AUD diagnoses reach as high as 36.7% in individuals 30–44 years old [3]. While the impact of AUD is clearly wide-ranging, the disorder remains poorly understood. Developing an understanding of the underlying biological mechanisms driving AUD is important for the development of novel treatments.

AUD is a complex phenotype characterized in the Diagnostic and Statistical Manual of Mental Disorders, 5th Edition (DSM-5) by multiple diagnostic criteria including alcohol consumption, craving, dependence, tolerance, withdrawal, and/or continued consumption despite negative consequences [4]. Both environmental and genetic factors play a role in each criterion and in the development of AUD [1]. Twin studies have shown heritability runs from 40 to 60% [5–8]. Differences exist in the cellular structure and gene expression in the brains of AUD patients [9–12] when compared to non-AUD control subjects. Whether these differences are purely the result of alcohol consumption, or due to preexisting differences in underlying structure, is unknown.

Strides have been made to understand the role that genetics play in AUD. In recent decades research has moved away from one-gene models towards models that encompass multiple contributing gene effects [1, 5, 13, 14]. This new understanding was facilitated by genome-wide association studies (GWAS) coupled with the fine genome mapping tools now available, and by transcriptional studies [14, 15]. However, human population studies are hampered by the long time course of AUD’s development and a patient’s unknown environmental factors. Because of this animal models of AUD are critical to furthering our understanding of the disorder. Modeling of AUD phenotypes across model organisms with well-characterized genomes has identified gene-networks that have provided novel insights [16–20].

In this review we will focus on lessons learned from genomic studies of alcohol behavior in *Drosophila melanogaster* (henceforth, the fly) and their implications for AUD. Flies share approximately 75% homology with the human genome in disease-causing genes [21–23] and representation of all major gene families found in humans, making them a cost-effective model for studying the role of genes and gene products in human disorders [24]. Flies also possess a smaller and less complex genome, as well as a smaller, more characterized nervous system that coordinates a broad repertoire of behaviors [25]. Because of this it is relatively easy to validate the behavioral roles of candidate genes, putting the fly model at the forefront of genomic studies of AUD. Additionally, the fly offers the potential to develop a mechanistic understanding of the genes involved in AUD phenotypes. To highlight the advantages of the fly for studies of AUD we will describe the techniques used to study alcohol genetics in flies and the specific gene networks involved in alcohol behavior that have already been identified there.

## Part 1: Techniques for studying alcohol genetics in flies

### Alcohol behaviors in the fly

Like humans, flies have a robust evolutionary relationship with alcohol. They hold a naturally evolved preference for laying eggs on fermenting fruit, and show many of the defining characteristics of AUD [24, 26, 27]. Compared to humans flies show increased resistance to many of the effects of different primary alcohols, including ethanol, the least toxic alcohol in mammals and the fly [28]. However, both humans and flies show natural variations in sensitivity to different effects of alcohol, including its stimulating effect at low doses, and its sedative effect at high doses [29, 30]. Similar to mammals, flies develop both metabolic and functional tolerances to ethanol, and show evidence of reinstatement after withdrawal [27, 30, 31]. Additionally, flies show robust preference learning when alcohol is paired with either olfactory, gustatory, and/or visual cues [32].

While the fly is well-suited for AUD studies, it is worth noting that to date flies only display a subset of phenotypes associated with the disorder. The most widely studied are alcohol sensitivity, alcohol tolerance, and preference learning.

### Alcohol sensitivity assays

Alcohol in low quantities produces stimulatory effects in both humans and flies [33–36]. At larger quantities it produces nervous system depression [28, 33, 37]. Sensitivity to these effects in humans is one of the strongest predictors of AUD; Schuckit et al. tracked men for 25 years and determined that baseline alcohol sensitivity was the greatest predictor of AUD [38]. Their findings suggested that those who were resistant to alcohol effects would require greater quantities of alcohol in order to experience the positive feelings associated with alcohol and thus were risking greater alcohol induced changes [39].

In the fly there are a number of ways to assess alcohol sensitivity phenotypes (Table 1). One of the earliest assays, the “inebriometer” [24, 40], assessed the disinhibitory effects of alcohol on large fly populations. Flies exposed to increasing concentrations of alcohol in a large vertical tube would gradually fall due to the loss of postural control and sedation (for a more detailed description see [24]) [41]. Flies will also become sedated following exposure to long-periods of low concentration alcohol vapor. The inebriometer assay has been refined to quantify subtle behavioral changes due to alcohol exposure, and to increase the efficiency of screening large numbers of genotypes at once. This upgraded assay is known as the “booz-o-mat” [35]. In it, flies are placed in horizontal tubes through which humidified alcohol vapor is passed. By placing the apparatus horizontally any pre-existing motor deficits in the flies are minimized as they no longer need to climb vertically or hold onto supports. This assay also provides tighter control over alcohol concentrations and finer analyses of fly behavior during and after alcohol exposure [35]. For example, in the booz-o-mat the stimulant effect of low doses of alcohol becomes apparent as an increase in locomotor behavior, as does the eventual decrease in activity (until full sedation) seen with higher levels of alcohol [35]. The sedative inebriation of flies in this assay reflects high levels of inebriation in the same way that the loss of righting reflex (LORR) does in mice [39]. Another measure of alcohol sensitivity is time to recovery following sedation. Flies are exposed to ethanol vapor of a given concentration (e.g. 35% ethanol of a cotton plug) until all flies are sedated, and then the time to recovery is recorded [19, 42].

**Table 1:** Definitions of important terms found in fly AUD research

Term:	Definition
ChIP-seq	Chromatin immunoprecipitation sequencing
GWAS	Genome wide association studies (also known as WGA—whole genome association)
MB	Mushroom body; an important neural center in flies; involved in olfaction and olfactory memory formation
NGS	Next generation sequencing (massive parallel sequencing)
RNA-seq	RNA sequencing (whole genome RNA sequencing)
SNP	Single nucleotide polymorphism
Wildtype	Aa model strain of organism fly with no induced mutations
RNAi	Short hairpin RNA used to target RNA for degradation. Used to knockdown expression of genes
Behavioral assays: terms	
booz-o-mat	Aa horizontal assay for intoxicating flies
Inebriometer	Aa vertical assay for intoxicating flies
CAFE	Ccapillary feeding assay
PER	Pproboscis extension reflex; used to study alcohol’s appetitive properties

Alcohol sensitivity is indicated by a population mean elution time (MET) in the inebriometer, or time to 50% sedated or recovered in sedation assays [19, 41–43]. The low cost and high-throughput nature of these assays make them very powerful for screening large numbers of flies in a relatively short period. Results from these assays demonstrate that flies may be evolutionarily adapted to prefer alcohol-rich environments and demonstrate robust behavioral responses to alcohol, and that these responses can change due to genetic and environmental manipulations [16, 44–46].

### Alcohol tolerance assays

Tolerance, the consumption of larger quantities of alcohol to achieve the same response or exhibiting a lower response to the same quantity of alcohol, is a defining characteristic of AUD. In many human studies alcohol consumption rates are used as the defining AUD phenotype for gene identification [9]. Flies can also develop the three types of tolerance to alcohol [24, 26, 28]: acute tolerance (a model for binge drinking), rapid tolerance (tolerance following metabolism of a single dose of alcohol), and chronic tolerance (developed from repeated alcohol exposure) [47]. The fly has been used to study these modes of tolerance in many ways. Findings include that larvae grown on media containing alcohol prefer higher alcohol concentrations as adults [48], adult flies who have consumed alcohol will consume alcohol in greater quantities during a second exposure [27, 47], and adult flies exposed to alcohol vapor are more resistant to alcohol’s behavioral effects upon second exposure [35, 47, 49].

The same behavioral assays described above for studying sensitivity are also used when looking at tolerance. In the inebriometer, higher METs are seen on the second exposure to the same concentration of alcohol vapor and recovery time from sedation decreases [43, 45, 50]. Studies performed using the booze-o-mat have also shown the development of rapid tolerance, particularly to stimulatory effects of alcohol upon second exposure [35, 51].

Consumption assays are also well-suited for studying alcohol tolerance. Long-term exposure is modeled through raising flies on alcohol-infused food [48, 52]. This type of assay not only models chronic tolerance but also is used to model fetal alcohol syndrome [53]. Other consumption-based assays involve short term access to alcohol-infused food [27, 54]. The capillary feeder (CAFE) assay can be used to quantify the amount of a liquid consumed by a population of flies in a given period. In this assay the amount of fluid consumed from filled capillaries is measured and compared to an evaporation control [54]. Alcohol exposure results in flies increasing their consumption of alcohol in this assay [44]. One notable precondition to this assay’s use is that in order for flies to drink to the relevant blood alcohol levels, they must first be starved prior to being placed in the CAFE assay [27, 44]. This induces a stress which is important to note in any discussion of gene networks involved in alcohol consumption in the fly [55]. Variations of the CAFE assay can create a choice between two capillaries containing different stimuli. This has been used to study alcohol tolerance as well as preference in the fly [44]. Flies will prefer to drink alcohol over water, and prefer to consume more alcohol following prior exposure to alcohol [27]. Flies show adaptation to both the negative and positive aspects of alcohol consumption, facilitating increased alcohol intake.

### Alcohol association preference assays

While the assays described above measure specific alcohol-induced motor or gustatory behavior, many others have been developed based on well-characterized learning and memory assays to assess the encoding of alcohol’s negative and positive valences [32]. The proboscis extension reflex (PER) assay models short term memory of gustatory cues [56]. As flies extend their proboscii in response to different nutrients, the rate of extension indicates learned associations with appetitive or aversive substances [57]. Like the CAFE assay described above, flies must also be starved prior to performing the PER assay, complicating its interpretation.

The most commonly used alcohol preference assay is based on the classic olfactory T-maze used in fly learning and memory studies. Flies are exposed to inebriating concentrations of alcohol while also exposed to a neutral odor [32]. Because the fly increases its proximity to appetitive odors, its movement towards or away from alcohol-paired odors indicates whether the fly perceives alcohol intoxication as appetitive or aversive, respectively [24]. Initially, alcohol induces an aversion to an odor paired with it, but later this reverses so the paired stimulus is preferred [32].

Recent work has detailed conditioned associations of alcohol at different concentrations and exposure times [58]. It has shown that low levels of alcohol do not form appetitive long-term cued memories, whereas moderate levels of alcohol form appetitive memories and these memories are enhanced by multiple cue pairings [58]. At high levels of alcohol exposure flies fail to form long-term appetitive memories and in some cases form long-term aversive memories, though there is evidence of short term attraction [58]. From this work it is clear that at different time points, and at different concentrations of alcohol, behavioral responses will vary. This work also points out that the most relevant alcohol concentrations, if one wants to model alcohol’s rewarding and cue-induced addictive qualities in the fly, are moderate doses around 8 mM spaced over multiple days, or between 6 and 8 mM trained on a single day with multiple exposures [58].

### Variations on alcohol conditioned learning assays

It is worth discussing two studies that used notably different methods to assay alcohol conditioned preference. Both assays include an element of stress, which is often neglected in conceptualizing alcohol behavior in the fly. Flies are known to experience stress, which changes their behavior in ways similar to mammals [59]. Stress is a major contributor to AUD, and particularly to relapse behavior [60]. These studies may provide novel insights into how ancient stress pathways interact with alcohol behavior, particularly with tolerance and reward.

Flies have evolved a mechanism to increase alcohol consumption to protect their larvae from parasitic wasps [61, 62]. Exposure to these wasps drives females to lay eggs on food sources with higher alcohol concentrations. Naïve flies prefer food with 3% alcohol for laying, whereas 6–12% is preferred in flies exposed to wasps [56, 62]. This level of alcohol is considered toxic due to the reduced viability of exposed offspring, but is effective in reducing wasp infection in the fly larvae [61, 63]. This demonstrates a strong drive to attain alcohol in wasp-exposed flies, even at toxic levels. This behavior is observed even after the wasps no longer pose a threat [62, 63]. However, the cues that drive the female to seek higher alcohol concentrations and the underlying transcriptional changes that facilitate retention of this behavior remains poorly understood. The stress of wasp exposure may be analogous to the stress of starvation necessary for flies to consume higher alcohol in the CAFE assay. Known regulators of fly anxiety-like behavior, including GABA, dopamine, serotonin, and octopamine (norepinephrine homologue) signaling are important in alcohol behavior [59, 64–69].

Another study linking alcohol intake to stress demonstrated that male flies who fail to copulate with a female increase their alcohol intake [70]. The authors argued that the reward of copulation could supplant the rewarding nature of alcohol. Another interpretation of this study is that failed copulation increases stress, leading to increased alcohol consumption. Work by Winbush et al. looking at transcriptional changes following 30 min of courtship (no copulation) supports the notion that courtship rejection is stressful [71]. In addition, Devineni and Heberlein demonstrated that flies will overcome aversive signals to seek alcohol, and these aversive signals also induce behavioral stress phenotypes such as increased wall following [27, 59]. Not only is there an increase in the behavioral stress response, but transcriptional changes following aversive stimuli indicate an increase in cellular stress response genes [72]. Other stressors are known to effect alcohol responses across species. One such stressor is sleep deprivation [73, 74]. Changes in sleep patterns often occur following environmental stressors, and these changes may also influence alcohol behavior. Recent work demonstrated overlapping pathways between alcohol sensitivity and sleep deprivation sensitivity through the adenosine A1 receptor [74].

### Genomics and transcriptomics

Beyond serving as an ideal model for alcohol behavior, the genetics of the fly is highly tractable. Over 600 different fly genomes are available for analysis (see section for wildtype populations of flies in Table 2) and over 4.8 million single nucleotide polymorphisms (SNPs) have been identified in the fly genome (dbSNP build 149) [75–78]. Together, the level of fine mapping and the availability of fully sequenced large populations of flies have made the fly an ideal model to study behaviors with complex genetic underpinnings.

**Table 2:** Tools and resources available to study AUD in the fly

Fly resources		Refs
Drosophila network resources	https://wiki.flybase.org/wiki/FlyBase:Drosophila_Network_Resources	
Flybase	http://flybase.org/	[178]
Bloomington Stock center	https://bdsc.indiana.edu/index.html	
Alliance of genome resources	https://www.alliancegenome.org/	
Wildtype populations of flies		
Drosophila genetic reference panel (DGRP)	http://dgrp2.gnets.ncsu.edu/	[77]
Drosophila synthetic population resource (DSRP)	http://wfitch.bio.uci.edu/~dspr/index.html	[75]
Global diversity lines	http://www.johnpool.net/genomes.html	[76]
Genetic tools for cell-type specific next-generation sequencing		
INTACT	Nuclear purification	[117]
TRAP	Translating Ribosomal Affinity Purification	[114, 115]
TU or EC tagging	Labels RNA following feeding of 4-thiouracil or 5-ethynylcytosine	[179, 180]
CAST-ChIP		[118]
DamID	Methylation-based chromatin profiling	[181]
Relevant collections of fly strains for behavioral validation		
Vienna RNAi lines		[182]
Transgenic RNAi project		[183]
FlyLight	https://www.janelia.org/project-team/flylight	[184]
Gene disruption project	MiMIC and CRIMIC lines http://flypush.imgen.bcm.tmc.edu/pscreen/about.html	[86]
Relevant drosophila transcriptional data sets		
Updated mapping of Drosophila sequence read archive	GEO Series GSE117217	
Single-cell whole brain	GEO Series GSE107451	
Kenyon cells	GEO Series GSE115718	
Kenyon cells	GEO Series GSE119629	
Kenyon cells and subset of MBONs	GEO Series GSE74989	
Mating behavior	GEO Series GSE104706	
Antennal lobe	GEO Series GSE99545	
CREB RNA binding	GEO Series GSE59611 GEO Series GSE73386	
Ethanol exposure RNA expression	GEO Series GSE77792 GEO Series GSE89137 GEO Series GSE48449	
Gene network analysis sites		
modENCODE	http://www.modencode.org/	[185]
SCENIC	http://scenic.aertslab.org	[186]
AmiGO 2	http://amigo.geneontology.org/amigo/	[187, 188]
DAVID bioinformatics database	https://david.ncifcrf.gov/	[189, 190]
MEME-ChiP	http://meme-suite.org/index.html	[191]
Weighted gene co-expression network	https://horvath.genetics.ucla.edu/html/CoexpressionNetwork/	[119, 192]
Reactome	https://reactome.org/	[193]
Cytoscape	https://cytoscape.org/	[194]
Geneweaver 2	https://beta.geneweaver.org/	[195]

The fly can easily be used to validate gene mechanisms in human disorders. A prime example of this is in the validation of a SNP in the intronic region of the human *AUTS2* gene. This SNP was found in a GWAS study looking at alcohol consumption [79]. The *AUTS2* gene was also identified when comparing a mouse line bred for high alcohol preference (HAP1) with low alcohol preferring mice (LAP1) [79, 80]. Mechanistic validation was demonstrated in the fly through reduced expression of the fly homolog *tay*, resulting in a blunted sensitivity to alcohol [79]. Tay, a negative regulator of the Epidermal Growth Factor Receptor (EGFR) pathway, has been implicated in a number of studies of alcohol behavior in the fly [16, 44, 45, 49, 81, 82] as well as mammalian studies (reviewed in [83]). Further mechanistic validation studies of mammalian genes implicated in AUD in the fly will be enhanced by large scale efforts underway, including the determination of the fly brain connectome [84], identification of each neuron molecularly and cellularly [85], creation of mutations in every gene in the fly genome [86], and identification of the transcriptional landscape across time, anatomical structures, and experimental conditions [72, 87–90].

The fly is also ideal for use in GWAS studies, where it allows for tight control over environmental factors. The Drosophila Genetics Reference Panel (DGRP) and, more recently, the Drosophila Synthetic Population Resource (DSPR) are comprised of inbred fly lines derived from wildtype flies over many generations [75, 77]. The DGRP consists of approximately 200 lines whose genomes have been sequenced [91]. Transcriptional information is now available for a subset of those lines [44, 77, 89, 91]. Further refinement of the DGRP is possible through an advanced intercross scheme that puts a diverse subset of DGRP lines through a mating routine which refines the areas associated with SNPs [44]. Both the DGRP lines and the advanced intercross lines have been instrumental in identifying gene networks associated with sleep, lifespan, olfactory behavior, and alcohol sensitivity and tolerance [16, 92–96].

GWAS studies have limitations, including the inability to predict transcriptional changes in response to alcohol exposure and chronic use [9, 15, 17, 80, 97]. Transcriptome analysis has shed light on this research area. Microarrays and whole transcriptome shotgun sequencing (RNA-seq) are the tools that have been most commonly used to perform transcriptome analysis (techniques reviewed by [98]). While microarray technology has come a long way over the last two decades, it is still restricted by the quantity of probes that can be put on a chip and by probe choice [98]. That said, RNA microarrays are still a particularly cost effective method to characterize responses to alcohol within specific brain regions, cell-types, and across species [10, 43, 49, 64, 80, 98–101]. In both RNA-seq and microarrays, sample quality is critical; particularly in microarrays due to their dependence on probes for transcript identification [98].

RNA-seq methods are now preferred over microarrays for whole genome profiling, as their greatest strength is the ability to uncover a range of different transcriptome complexities without reliance on probes. This is particularly useful when considering potential alternative splice forms and non-coding RNAs associated with AUD [102–104]. Alternative splice forms have been shown to play a role in alcohol behavior in humans and flies [105–107]. It has been established that alternative splicing of the *slowpoke* gene mediates fly alcohol tolerance [40, 108] and the full impact of alternative splicing events is only now being appreciated. Signor and Nuzhdin recently made available a comprehensive data set from a long-read RNA-seq study [107]. This work showed that changes in exon expression due to alcohol presentation build the longer animals are exposed [107]. Limited alternative splicing in response to alcohol (meaning similar expression of gene but alternative exon expression) was shown, but changes in the rank order of the presence of specific exons changed based on alcohol presentation [107].

Recent human transcriptome studies have shown the impact of long-term alcohol use on subtle transcriptional changes in multiple brain regions, including the nucleus accumbens, hippocampus, and prefrontal cortex [9, 97, 99, 109]. These areas are important for reward, learning, and executive functioning and are strongly implicated in AUD [65]. Within the hippocampus RNA-seq work has identified potential neural adaption mechanisms important in alcohol’s addictive properties [97, 109]. Recent work has also shown evidence of alternative gene splicing across different brain regions, emphasizing the need for more region-specific and cell-specific studies [110].

One powerful technique that allows investigation of region- and cell-specific transcriptional changes in the fly is the GAL4/UAS system [111–113]. This technique allows the yeast GAL4 transcription factor to be expressed under the control of a specific gene promoter or enhancer. The GAL4 transcription factor binds to an upstream activating sequence (UAS), which leads to the expression of a downstream gene [111]. Large collections of GAL4 and UAS fly lines exist (see Table 2). Using the GAL4/UAS system fly geneticists can express almost any protein in any cell type, allowing cells to be identified and sorted based on fluorescent protein expression or tagged proteins. These tags can be used to perform cell-type specific transcriptional analysis by a variety of techniques, including translating ribosome affinity purification (TRAP) [114, 115], isolation of nuclei tagged in specific cell types (INTACT), and chromatin affinity purification (CAST-ChIP) [116–118] (see Table 2 for additional tags).

These transcriptional analysis techniques can distinguish transcript changes due to stimuli versus those due to network regulation. Many genes work within networks where a change in expression in one gene may actually reflect a change in an entire network of genes that share common regulators. Network analysis can find hubs in gene networks which can point to common regulatory networks (see weighted gene coexpression network analysis (WGCNA)) [119]. It can also identify novel regulatory mechanisms including epigenetic modifications that cause coordinated gene expression changes. These epigenetic modifications play a role in alcohol behavior across species [19, 120]. Modern sequencing techniques like ChIP-seq have greatly increased our ability to directly study the epigenetic changes that occur during alcohol behavior. This method utilizes antibodies for specific transcription factors or chromatin modifiers to tag DNA where transcription is potentially occurring [121].

Collectively, these genomic and transcriptomic techniques have provided information on both the underlying genomic landscape as well as changes to this landscape that drive alcohol behavior in the fly.

## Part 2: Gene networks implicated in alcohol behavior in the fly

Genomic and transcriptomic studies of alcohol behavior in the fly have provided insight not only into the individual genes that play a role, but also into their functional networks. Over the last 20 years our understanding of how these networks fit into the behavioral mechanisms of alcohol sensitivity, tolerance, and conditioned preference learning has greatly expanded. This is partly due to increased gene annotation, increased availability of data sets, and public databases that compare across studies and species (see Table 2 for a list of relevant datasets). These genomic survey studies have reinforced the findings of earlier forward genetic screens that identified single genes involved in alcohol behavior (see reviews [24, 31, 52]) and are now shedding light on how these genes act in the networks identified more recently [82, 106, 122].

Morozova et al. have demonstrated the involvement of networks regulating metabolic enzymatic activity, oxidative stress, glial functioning, nervous system functioning, metabolism, chromatin modification, cytoskeleton dynamics, and immune response genes in alcohol behavior [16, 43, 45, 53, 123]. In addition, they found that distinct genes affect alcohol sensitivity and alcohol tolerance [16]. Extensive review of the role metabolic enzymes and neuronal signaling genes play in alcohol behavior can be found elsewhere [31, 52, 124]. We will now highlight gene networks identified in the fly that have received less attention and will be crucial in understanding alcohol behavior across species [18, 100, 125–128].

### Chromatin remodeling genes

Chromatin remodeling in the form of histone acetylation, deacetylation, methylation, and demethylation all play a role in transcription by modulating access to the DNA. Genes coding for chromatin remodeling enzymes are important in the discussion of gene networks, not only because they have been implicated through gene network analysis in alcohol behavior across species [19, 51, 97, 109, 120, 129–131], but because they possess the capability to coordinate gene networks. Histone acetylation via histone acetyltransferases (HATs) can provide access to DNA in a coordinated and long-lasting fashion, resulting in a network of transcripts becoming upregulated, while histone deacetylases (HDACs) mediate the opposite function.

Ghezzi et al. exploited the coordinating functions of HATs and HDACs using ChIP-seq methods to probe for histone acetylation changes due to alcohol tolerance (both benzyl and ethyl alcohol) [19]. Targeting the histone H4 acetylation sites, due to their associations with active transcription, they identified a series of coordinated gene expression modules across different alcohol exposures and from other environmental stimuli such as heavy metals. Among these modules was a set of genes enriched for ion channels and synaptic proteins. Interestingly, they found genes with alternative splicing known to play a role in alcohol behavior: *Dscam* and *slo* [107, 108]. Ghezzi et al. validated *slo*’s role in alcohol tolerance, but *Dscam* manipulation did not influence the behavior. Importantly, their validation was done using an RNAi knockdown fly line for *Dscam* which may not have targeted the correct transcript variant [19]. This underscores the importance of transcript variants in alcohol behaviors, and these results remain to be verified in follow-up analyses. This study also found that H4 acetylation at HDAC genes was occurring in response to tolerance. *HDAC6* not only showed increased acetylation, it also showed increased transcript expression across different environmental stressors, with the highest expression in response to heavy metals [19]. Thus, alcohol appears to increase expression of a coordinated network of genes to promote tolerance, and one of these genes coordinates the silencing of other genes.

*Sir2*, the fly homolog for mammalian *Sirt1*, is another HDAC gene that has proven important for alcohol sensitivity, conditioned preference, and the development of tolerance [43, 49, 51]. In mammals, Sirt1 in the brain is directly involved in neurogenesis, dendritic morphology, plasticity, regulation of micro RNAs (miRNAs), energy metabolism, and circadian rhythms [132]. It is down-regulated following alcohol exposure in the fly [43, 133]. Alcohol tolerance in the fly depends upon expression of *Sir2* in the mushroom body (MB, a neuropil important for learning and memory in the fly [134]) [32, 51, 135]. Engel et al. also demonstrated that the loss of *Sir2* disrupted upregulation of approximately 90% of genes that responded to alcohol exposure [51]. This implies a very different transcriptional landscape without *Sir2*. Part of the difference was in the expression of pre-synaptic proteins. In order for tolerance to develop synaptic reorganization in the MB needed to occur. This was *Sir2* dependent. Taken together, since histone acetylation changes the long-term expression of genes, exposure to alcohol may effect tolerance by altering histone acetylation. Evidence for this model was supported by Adhikari et al. who showed that *Hr38* expression needs to be terminated by *Sir2*, which leads to a reduction in *Hr38*, which in turn results in an increase in tolerance [136]. *Hr38* is also down-regulated 7 h following wasp exposure [63], pointing to ongoing silencing of this gene to promote tolerance and alcohol preference.

### Glial affiliated genes

Glial dysfunction and structural change as a consequence of alcohol consumption have been documented across species [100, 127] and glial involvement in alcohol behavior in the fly is not new. Early forward genetics screens showed disruptions in the blood brain barrier (BBB) leading to alcohol resistance [137]. *Moody*, an orphan G-protein coupled receptor, compromises the BBB due to its role in maintaining tight septate junctions in glial cells. This causes decreased alcohol sensitivity [137]. While *Moody* has not appeared in genomic survey studies, its regulator, *loco*, has [44]. Both *moody* and *loco* are also important in cocaine sensitivity [137].

Cytoskeletal proteins have also been implicated in glial functioning in response to alcohol, particularly those involved in actin cytoskeleton organization, which are upregulated following alcohol exposure [49]. Recent work has found evidence for actin dysregulation resulting in major topology changes in the perineurial glia (the flies BBB) that persist over 24 h, which may be a mechanism that leads to tolerance [122].

In addition to these genes, whole brain analysis has identified a number of other glial related genes that are involved in in alcohol sensitivity and consumption. These include the genes *drpr*, *pbl,* and *fas* [44]. Of particular interest is *Draper* (*drpr*), which increases expression in response to glial cell injuries [138]. *Draper* codes for a cell surface receptor found on ensheathing glia in the adult brain, and is involved in the glial phagocytic response following injury or cellular damage [139]. *Draper* is also important for synapse reorganization (including in the mushroom body) [140], which is hypothesized to underlie tolerance and alcohol conditioned place preference [16, 106, 124, 141, 142]. This hypothesis has been supported by the findings in genomic survey studies that identify synaptic signaling and synaptic structural proteins play a role in alcohol behavior [43, 44, 49, 143]. Synaptic reorganization is necessary for the development of tolerance and conditioned place preference in the fly [31, 51, 141, 142, 144]. Subtle underlying changes in *Draper* function may make synaptic reorganization, which facilitates behavioral extinction, harder following initial cue-reward learning. This may be why *Draper* appears in GWAS studies but has not appeared in prior transcriptional studies.

The role of glial signaling in alcohol behavior is under-studied in the fly and may provide a mechanistic understanding of the role alcohol plays in glial functioning. Recent findings from the mammalian nucleus accumbens using single cell analysis in response to opioids aligns with the hypothesis that glia help shape rewarding memories [145].

### Cellular stress genes

Cellular stress genes present in glia and/or neurons also play a role in alcohol behavior across species [18, 49, 50, 60, 126, 128, 146]. Genes involved in oxidative stress, heat shock, and unfolded protein responses routinely appear in gene networks for alcohol behavior [128, 147]. Kong et al. found multiple heat shock proteins up-regulated following alcohol exposure. *Hsp 22, Hsp70Ba, Hsp 70Bc, Hsp70Aa, Hsp23, Hsp26, Hsp68, Hsp27* all show differential expression following alcohol sedation [49]. The stress of a heat shock assay itself can induce tolerance through a *hangover* gene-dependent pathway [50]. Both heat shock and cold shock show overlapping gene expression with alcohol exposure as well [19]. Previous work also implicated *Hsp70Aa* as an immediate response gene to alcohol presentation [49]. However, these transcriptional studies were done on whole fly heads. The specific cells in which these genes networks mediate alcohol responses remain unclear.

Genes involved in oxidative stress have also been implicated in alcohol behavior [146, 148]. Most notably the gene *jwa*, which is upregulated in mammals in response to oxidative stress and heat stress [148], is important for the development of tolerance [148]. Other relevant stress response genes implicated in alcohol phenotypes and showing differences in expression levels in response to alcohol are *msn*, *Mpk2* and members of the cytochrome P450 family [19, 44, 49, 53]. These differences in gene expression of heat shock proteins and other cellular response genes (especially for environmental toxins) in response to alcohol exposure may reflect alcohol’s cellular toxicity at high doses. Hence, consistent with the glial work described above, increased cellular stress in both glia and neurons facilitate cellular changes that reduce fly sensitivity to alcohol. Indeed, in the case of *msn*, RNAi-mediated knockdown of the gene throughout the animal resulted in decreased sensitivity to alcohol’s effects on viability [53], but restriction of the knockdown to neuronal populations showed no effect on tolerance [19].

### Innate immune response genes

Innate immune response genes are often found in the same gene networks as cellular stress genes. Multiple studies examining transcriptional changes following alcohol exposure in the fly have found changes in innate immune response genes [49, 143, 149, 150]. Kong et al. found up-regulation of both the Toll and Imd pathways [49]. These pathways converge onto and regulate nuclear factor-κB (NF-κB), a critical factor in the innate immune response. A number of these genes are implicated in alcohol sensitivity in other studies as well, including *cact, rel, dnr1, dro5,* and *Spn27A* [43, 143, 149]. More recent work on the Toll signaling pathway in alcohol behavior demonstrated that reducing the activity of this pathway through mutation or RNAi knockdown increased sensitivity to the sedative effects of alcohol [149]. Increasing Toll pathway activity through overexpression led to decreased sensitivity to alcohol sedation [149]. Activity of the Toll pathway is conserved across species in response to alcohol [147] (reviewed by [151]).

The gene *Spn27A* is also known to be involved in the innate immune response, where it is upregulated following bacterial infection [152]. It is increased in the fly on the same order of magnitude in response to alcohol [49]. Reduced levels of *Spn27A* result in increased sensitivity to alcohol, but minimal differences in tolerance [49], similar to the behavioral response seen with manipulations to the Toll pathway [149].

While these genes may not play a role in tolerance, evidence suggests that they may be triggers for the alcohol-based wasp defenses described previously. Innate immune response genes are one category of gene that show increased expression following the shift in alcohol preference in the presence of wasps [63]. Based upon the findings from the Toll pathway, activation of immune pathways in response to wasps is expected to increase resistance of the flies to alcohol, allowing them to sustain themselves on higher alcohol content food without the locomotor or sedating effects of the drug. Increased alcohol concentrations within the fly also prevent bacterial infection. So in the presence of infection, changes in gene expression allow flies to sustain themselves on higher alcohol food.

### Brain structure- and circuit-level analysis of gene networks

While the mechanistic relationship between these gene networks and changes in alcohol behavior is poorly understood, the fly presents a critical avenue for further research. Many of the components of the networks outlined above were validated through mutant analysis or RNAi-mediated knockdown of targeted genes (for a listing of comprehensive collections of fly mutants, RNAi fly lines, and GAL4 fly lines in which to express RNAi lines, see Table 2). While most have been validated using whole-body mutants or RNAi knockdown in all tissues [19, 44, 45, 49, 53, 150], a number of genes have been validated in neuronal tissue using the *elav*-GAL4 line [19], in glial tissue using *reaper*-GAL4 [122], or in even more specific brain structures such as the MB [106] and the ellipsoid body [153]. Utilization of the GAL4/UAS system to validate genes in specific brain structures or circuits is an important advantage of the fly.

There is a long history in the fly of overlap in the genes important for learning/memory, alcohol behavior, and the reward system (reviewed by [124, 154]). One of the first genes discovered to show an alcohol phenotype was *amnesiac*, already extensively studied for its role in aversive olfactory memory through an MB-dependent circuit [155–157]. The MB plays a role in both tolerance following ethanol exposure and in ethanol-induced hyperactivity in flies [51, 144]. It is also necessary for the expression of alcohol conditioned preference [32], and it functions as a dopaminergic integration center and epicenter for associative learning, particularly in the formation of olfactory memory [134]. The brains of flies only contain approximately 130 dopaminergic neurons, of which a subset panel the MB structure. This dopamine system regulates the flies’ reward signaling, short- and long-term memory formation, aversive memory formation, wakefulness, foraging behavior, and oviposition preference [66, 158–164]. Thus, dopaminergic signaling and modulation of the MB circuit is a crucial component in the development of associative ethanol memories, as well as the encoding of aversive and appetitive ethanol memories [32, 106, 164]. While this work has been primarily accomplished through forward genetic screens, evidence from GWAS studies exists as well. Work by Morozova et al. identified SNPs near the dopamine decarboxylase gene associated with variation in alcohol sensitivity [123]. This implies that underlying differences in dopamine signaling alters alcohol behavior.

As mentioned above, *Sir2* expression is required in the MB for normal alcohol tolerance to develop. Engel et al. demonstrated that Sir2 was important for determining the valence of alcohol and synaptic functioning in the MB [51]. More recent work using alcohol conditioned preference assays points to the actions of Notch signaling within the MB circuit for mediating long-term alcohol reward memories [106]. Morozova et al. identified members of the Notch signaling pathway involved in alcohol sensitivity [45, 150], and previously Kaun et al. found a role for *Scabrous* (*Sca*, a mediator of Notch signaling) in alcohol-associated preference [32]. Upon further exploration of the Notch pathway, Petruccelli et al. found that Notch and Suppressor of Hairless (Su(H)) signaling mediated by Sca in the MB is important for long-term alcohol preference [106]. Not only was this pathway important in the adult fly for memory formation, but Su(H) changes the transcriptional dynamics of the MB. Alternative splicing of *DopR2*, a dopamine receptor previously implicated in alcohol behavior [133] and AUD [165], occurs following alcohol exposure driven by Su(H) activation.

The Jak/Stat Innate immunity pathways also shows extensive crosstalk with the Notch pathway [166, 167]. *Stat92E* was found to be differentially expressed in rejected male flies. One isoform was up-regulated while the other was down-regulated 24 h post-rejection [71]. Recent work by Petruccelli et al. identified *Stat92E* as also being alternatively spliced within the MB following alcohol exposure [106]. Modifications in *Stat92E* levels due to alcohol exposure in the MB may underlie long-term changes in alcohol preference, as well as larger memory systems involving stress. Other genes involved in innate immune response and cellular stress have also been found in the MB [72]. Further work will need to be done to understand the roles innate immune genes in the MB play in memory formation.

### Caveats and considerations from gene network analysis in fly alcohol behavior

Studies of alcohol behavior genetics are confounded by the differing modes of alcohol exposure used [16, 44, 123]. If flies consume alcohol from their food source, genes enriched for feeding behavior and alcohol behavior are both enriched [44]. To illustrate, recent work using the DGRP lines have evaluated the overlap between food consumption and alcohol consumption [168]. The authors argue that among the genes associated with alcohol consumption, about 30% are associated with the caloric nature of alcohol [168]. Additionally, enrichment for olfactory genes is found when animals are exposed to alcohol vapor (either from a food source or air stream) [123]. A number of studies have demonstrated a role for olfactory genes in alcohol sensitivity and tolerance [49, 142, 150, 169]. *Lush*, one of the most characterized among these genes, encodes an odorant-binding protein known to bind to alcohols [169]. It is down-regulated in response to alcohol and is implicated in alcohol behavior [43, 49]). Additionally, recent work has shown that synaptic alterations driven by *Dunc13* (homolog of mammalian *Munc13*) in olfactory receptor neurons of the fly play a role in mediating tolerance [142]. Alcohol vapor-induced changes in olfactory genes were addressed by Kong et al., who explained that alcohol is toxic to the antennae cilia causing degeneration of the cilia [49]. Down-regulated olfactory genes are a response to this toxicity, whereas the upregulated olfactory genes may be involved in alcohol behavior. Recent work has also pointed to potential novel roles for olfactory associated genes outside of olfactory receptor neurons [72].

The use of differing modes of alcohol exposure across studies may help explain the lack of significant overlap in their identified genes (excluding functional gene networks which do show overlap). Even across studies using the DGRP lines very few overlapping genes are found. Study methods often differ not only in the mode of alcohol exposure but in length of exposure, concentration, time following exposure, and general experimental design and analysis including the bioinformatics tools used. One of the most surprising examples of this lack of overlap came from Troutwine et al., who used RNA-seq in an attempt to verify innate immune response genes, found by Kong et al. using microarrays [49, 149].

An additional concern is that while both GWAS and transcriptional methods seek to identify genes, the genes identified through GWAS are participating in the predisposition for an alcohol phenotype, whereas transcriptome studies are well-suited to identify changes in response to alcohol. For instance, significance in a GWAS study does not necessarily mean there will be a transcriptional difference in that same gene in response to alcohol. This is why looking at functional and coexpression gene networks may be more appropriate for studying alcohol behavior in the fly. Morozova et al. observed that if they loosened their cutoff accounting for multiple testings they picked up additional gene overlaps with other studies [16, 43]. If genes are working in coordinated networks and are not changing independent of each other then there should be a reevaluation of how we identify meaningful transcriptional changes.

One additional caveat when considering work done with the DGRP fly lines is that alcohol behavior is sexually dimorphic [44, 170]. Different SNPs are associated with alcohol behavior depending upon the sex of the fly [44]. Up to this point studies producing transcriptional data have either not found sexual dimorphisms or have not considered them when analyzing data. A number of studies only used male flies, including Morozova et al. [43], Kong et al. [49], Engel et al. [51], and Signor and Nuzhdin [107]. Troutwine et al. used only females [149]. Ghezzi et al. [19] and Urizar et al. [143] used mixed male and female populations. Additional transcriptional work conducted using controlled populations of both males and females must be performed.

## Part 3: Future directions

Morozova et al. elegantly demonstrated that identifying gene networks in the fly could inform human GWAS studies [170]. They used a candidate gene approach in humans for the genes identified in fly networks with homologs. Quantitative Trait Transcript (QTT) analysis for alcohol sensitivity (second exposure) showed a cluster of transcripts involved in metabolic enzyme activity. Centered in the fly network is the gene *Men* (*Malic Enzyme*), which has been identified previously in alcohol sensitivity across species [43, 171]. Targeting the human homolog *Malic Enzyme* (*ME1*) in the Framingham Heart Cohort showed small yet significant effects of SNPs on cocktail drinking [170]. This demonstrated the utility of identifying gene networks in flies and then using this information to inform the human data. As new gene networks are described, this type of cross species validation will prove insightful.

Further identification of coordinated gene networks through RNA-seq and ChIP-seq will shed light on larger pathways that could be targets for pharmacological treatment. In 2006 Riley et al. demonstrated that the human homologue of the *Drosophila hangover* gene is associated with alcohol dependence within an Irish cohort [172]. Both *hangover* and its human homologue *ZNF699* are zinc-finger proteins, making them potential coordinators of gene networks, and thus targets for future drug development. *Hangover* also plays a role in response to oxidative stress, further implicating the role of cellular stress genes in alcohol behavior [50, 173]. Other potential targets are the proteins regulated by Protein Kinase C (PKC). Recent work demonstrated naltrexone’s ability to block alcohol preference in the fly through PKC mechanisms [174]. Previous work implicated the PKC gene *inaC* in a network of genes associated with tolerance [170]. Both of the fly’s genes coding PKCs (*InaC* and *Pkc98E*) are also implicated in alcohol sensitivity via the Neuropeptide F (NPF) and serotonin circuits [175].

A gene network approach also illuminates developmental pathways still in use in the adult fly for mediating behavior [16, 32, 106], which were underappreciated until recently. Additionally, the roles of glia, cellular stress genes, and immune response genes in adult fly behavior are only now being fully realized. Further investigation is needed into the mechanisms that these pathways use to drive alcohol behavior. It is also clear from these genomic surveys that responses to alcohol change over time, both in the genes expressed and in splicing differences [49, 63, 107], which are only now being fully appreciated and will require further investigation.

Recent advances in proteomic research in the fly will also allow researchers to validate expression differences seen at the transcriptome level [176]. Recent proteomic analysis of postmortem human brain tissue of AUD patients showed changes in metabolic, trafficking, cytoskeletal, and excitotoxity proteins [177]. A significant amount of mRNA never becomes protein, so proteomic analysis may be a better method for examining the large number of genes with expression changes [89]. For example, recent work on proteomic changes in the fly brain following olfactory learning implicated the Jak/Stat pathway [176]. Large-scale proteomic studies in the fly will also identify novel networks of proteins associated with alcohol behavior.

## Concluding remarks

In the era of ‘Big Data’ the fruit fly is an advantageous model organism to use to acquire novel insights into alcohol behavior. Through the use of a broad set of genetic tools, well-characterized behavioral assays, and an ever-growing number of genomic survey studies, one can begin to piece together a mechanistic understanding of the genes involved in alcohol behavior. This work shows translational potential to understand some of the features of AUD in humans. In particular, recent work on learning mechanisms and tolerance in the fly have shown overlap with glial, cellular stress, innate immunity, and chromatin modifying genes in AUD. These gene networks point to a mechanism in which alcohol tolerance and learning is partially mediated through homeostatic mechanisms to deal with cellular injury. Further study of these gene networks in flies may provide a better mechanistic understanding of AUD and drug targets.

## Abbreviations

AUD: Alcohol Use Disorder
NESARC: National Epidemiologic Survey on Alcohol and Related Conditions
DSM-5: Diagnostic and Statistical Manual of Mental Disorders, 5th Edition
GWAS: genome-wide association studies
LORR: loss of righting reflex
MET: mean elution time
CAFÉ: capillary feeder
PER: proboscis extension reflex
SNP: single nucleotide polymorphism
HAP1: high alcohol preference mouse line 1
LAP1: low alcohol preference mouse line 1
EGFR: Epidermal Growth Factor Receptor
DGRP: Drosophila Genetics Reference Panel
DSPR: Drosophila Synthetic Population Resource
UAS: upstream activating sequence
TRAP: translating ribosome affinity purification
INTACT: isolation of nuclei tagged in specific cell types
ChIP: chromatin immunoprecipitation
WGCNA: weighted gene coexpression network analysis
HAT: histone acetyltransferase
HDAC: histone deacetylase
miRNA: micro RNA
MB: mushroom body
BBB: blood brain barrier
NF-kB: nuclear factor kappa B
QTT: Quantitative Trait Transcript
PKC: Protein Kinase C
